# Development of Eco-Mortars with the Incorporation of Municipal Solid Wastes Incineration Ash

**DOI:** 10.3390/ma16216933

**Published:** 2023-10-28

**Authors:** Inês S. Vilarinho, Gonçalo Guimarães, João A. Labrincha, Maria P. Seabra

**Affiliations:** CICECO—Aveiro Institute of Materials, Department of Materials and Ceramic Engineering, Campus Universitário de Santiago, University of Aveiro, 3810-193 Aveiro, Portugal; goncaloguimaraes@ua.pt (G.G.); jal@ua.pt (J.A.L.)

**Keywords:** bottom ashes, industrial waste, circular economy, sustainable construction materials, freeze–thaw resistance tests

## Abstract

The cement sector is the second largest contributor to anthropogenic CO_2_ emissions, and several efforts have been made to reduce its environmental impact. One alternative that has gained interest in recent years involves the use of municipal solid waste incineration (MSWI) bottom ash (BA) as clinker/cement replacement. This paper studies the application of MSWI BA in three different ways: (i) aggregate (0 to 100 *v*/*v* %), (ii) partial binder substitute (0 to 30 *v*/*v* %), and (iii) filler (5 *v*/*v* %). It stands out for its approach in characterizing seven distinct BA particle sizes and for the development and analysis of eco-cement mortars with only mechanically pre-treated BA. Hardened state properties showed that the use of BA as aggregate leads to deterioration and efflorescence formation on the surface of the mortars, making this application unfeasible. The replacement of 15 *v*/*v* % of OPC (Ordinary Portland Cement) by BA and the use of finer (<63 μm) BA as filler caused a decrease in the compressive strength of the mortar, from 15.8 to 9.3 and 11.0, respectively. However, these materials are suitable for use in walls where the minimum required mechanical resistance is 5 MPa. Furthermore, these mortars demonstrated resilience against freeze–thaw cycles and even exhibited increased compressive strength after 25 cycles. Thus, this work showed that MSWI BA can be used as an OPC substitute (up to 15 *v*/*v* %) and as a filler, promoting circular economy principles and reducing CO_2_ emissions related to the construction industry.

## 1. Introduction

Industrial activity is still responsible for the highest anthropogenic CO_2_ emissions, reaching, in 2021, 9.5 Gt of CO_2_ [1]. To counteract the evolution of greenhouse gas emissions, a Net Zero Emissions (NZE) scenario was developed to show the pathway to achieve net zero CO_2_ emissions by 2050. According to this scenario, CO_2_ emissions should fall to approximately 7 Gt of CO_2_ by 2030; however, progress is occurring very slowly [1]. Among the different industrial sectors, the cement industry is responsible for 27% of global CO_2_ emissions, being the second highest contributor [1]. Herein, the direct CO_2_ intensity increased by 1.5% per year between 2015 and 2021, but to align with the NZE scenario, an annual decrease of 3% is necessary [2]. One of the key areas for achieving this goal is to reduce the clinker-to-cement ratio. Currently, fly ash from coal power plants and ground granulated blast furnace slag from the steel sector is widely used; however, owing to the decarburization of other sectors, the availability of these industrial by-products is expected to decrease, and alternatives should be found [2].

Several scientific developments have been made to replace clinker/cement with other materials, such as calcinated clay and residues/by-products from other industries, i.e., paper pulp wastes [3]. One residue/by-product that has gained interest in recent years is the ashes resulting from municipal solid waste incineration (MSWI) [4,5,6,7,8]. Worldwide, approximately 2 billion tons of municipal solid waste are generated annually, and at least 33% of it is not safely managed [9]. In Europe, 236 million tons were generated in 2021, of which approximately 23 wt.% was landfilled, 26 wt.% incinerated, 30 wt.% recycled, and 19 wt.% composted [10]. The main advantage of the incineration process is the decrease in solid waste quantity (up to 70%) and volume (up to 90%), allowing, in some cases, energy recovery. However, residues are still generated, namely, fly and bottom ashes, which require proper disposal and utilization. To overcome this problem, the use of MSWI residues as secondary construction materials has been studied because the components present in the fly and bottom ashes are very similar to those of Ordinary Portland Cement (OPC) and aggregates [4]. MSWI ashes are described as heterogeneous solid granules with a wide particle size distribution, and, normally, to apply these materials, several pre-treatment methods are employed [4]. These treatments might include mechanical, thermal, and chemical processes, i.e., leaching of materials, such as chlorides and alkalis, generating more waste and increasing the cost of the process due to the use of chemicals, water, and energy [5].

Tang et al. [11] investigated the effect of grinding and thermal treatments on the properties of materials that use MSWI bottom ash (BA) with reduced particle size (0–2 mm) as a binder substitute. The authors substituted 30 wt.% of cement by these ashes and concluded that: (i) the addition of grounded BA, without any additional pre-treatment, delays the cement hydration reaction due to the presence of organic matter and heavy metals, but it was minimized when BA was subject to heat treatment; (ii) after 28 days of curing the mortars exhibited a mechanical resistance between 30 and 51 MPa, lower than the reference sample (61 MPa); and (iii) thermal treatment has efficiently reduced the leaching of compounds such as Sb, Cu, and chlorides.

Chen et al. [12] evaluated the influence of BA particle size on the hydration and microstructure of hardened pastes. The results showed that the chemical composition of BA was highly dependent on the particle size interval. The fraction with larger size (>1.8 mm) particles displays high silica content that can have a pozzolanic activity, while the smaller particles (<0.075 mm) contain calcareous materials and anhydrite, which showed a stronger retarding effect. Furthermore, this fraction also leached heavy metals, which prohibit the dissolution of C_3_S and delayed hydration reactions. However, after the onset of accelerated hydration, the authors observed that the different fractions of BA had similar hydration heat evolution, total heat of hydration, and cementitious properties.

Irshidat et al. [13] studied the feasibility of recycling MSWI ashes as a substitute for traditional aggregates in the production of cementitious binders. The compressive strength was enhanced with replacement rations of 10 and 20%. However, high concentrations of heavy metals (e.g., Cu, Cr, Mn, Ni, Zn, and Pb) were present in the leachate of mortars containing MSWI ashes, which could hinder any possible application.

Tokgoz et al. [14] studied the potential utilization of MSWI ashes (boiler and residue ashes) as supplementary cementitious materials in cement-based composites (replacement ratios of 0–30 wt.% of cement). The highest compressive strength was obtained with 10 wt.% replacement ratio of residue ashes. The boiler ashes induced a decrease in the mortars’ compressive strength, attributed to the presence of unburned carbon content. Leaching tests indicated that most toxic metals and salts, except Ba and Cl^−^, were stabilized in the cement matrix.

This study aims to investigate the application of MSWI bottom ashes in three different ways: (i) aggregate, (ii) partial binder substitute, and (iii) filler. For this purpose, seven particle sizes of BA were characterized. The novelty of this work is the development and thorough analysis of eco-mortar materials, incorporating only mechanically pre-treated BA. The hardened state properties of the mortars with two curing conditions (climatic chamber and immersion in water) were evaluated after 7 and 28 days of curing, and freeze–thaw resistance tests were performed.

## 2. Materials and Methods

### 2.1. Materials

The binder used in this work was “CEM II/B-L 32.5 N” cement, which is a limestone-based Ordinary Portland Cement (OPC). As an aggregate, calibrated commercial sand provided by Saint-Gobain Weber (Aveiro, Portugal) was used. Bottom ash (BA) resulting from the incineration of Municipal Solid Waste (MSW) at the ValorSul energy recovery station in Loures, Portugal, was used. This residue was supplied by the company already sieved (<5 mm). Further, in all formulations, distilled water was used.

The BA was pre-treated before use and underwent the following steps:

(i) Drying in an oven at 105 °C for 24 h;
(ii) Grinding in a “Retsch SK1” hammer mill until reaching a granulometry lower than 2 mm;
(iii) Sieving with different mesh openings: 63 μm, 125 μm, 0.5 mm, and 2 mm.

**Figure 1:**
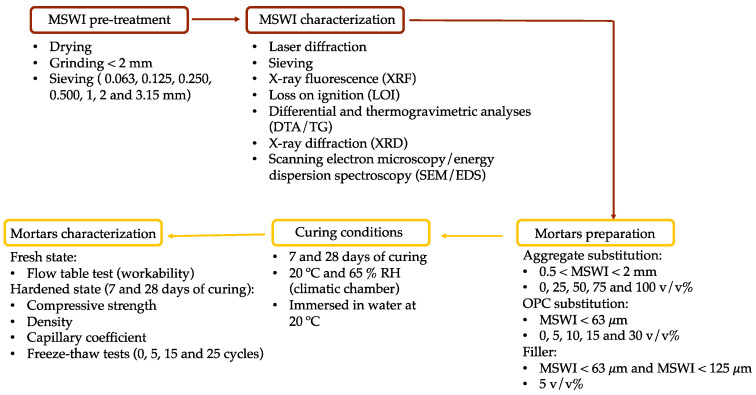
Flowchart of the experimental procedure.

The smaller fraction (<63 μm) was utilized as a substitute for OPC and also as a filler. The fraction with particles between 63 μm and 125 μm was used as filler, while the fraction between 0.5 and 2 mm was utilized as aggregate. Figure 1 presents the flowchart of the experimental procedure, including the MSWI pre-treatment and the characterization techniques used, the mortar preparation, curing conditions, and respective characterization methods.

### 2.2. Preparation of the Eco-Cement Mortars

The prepared formulations are presented in Table 1. The alphanumeric reference of the formulations portrays how the ash was integrated into the mixture and the amount used. In formulations with the reference B_x_, the binder (OPC) was partially replaced by BA (fraction < 63 μm), while in A_x_, the aggregate was replaced by the ash (fraction between 0.5 and 2 mm). In both situations, x indicates the percentage (by volume) of BA used. In formulations where BA was used as a filler (fraction below 63 μm and between 63 and 125 μm), the incorporated percentage was always 5 *v*/*v %*. Thus, the reference for these formulations is F_5–y_, where y refers to the sieve mesh opening used in the pre-treatment (63 or 125 μm).

In the mixtures, the ratio (in volume) of aggregate/binder (A/B) was kept constant (A/B = 3). However, to maintain the workability of the paste constant, the water/binder (W/B) ratio used in each formulation was adjusted to achieve a final spread value of 140 ± 3 mm.

The procedure used in the preparation of the mortars consisted of the following steps:

(1) Homogenization of the solid components (binder, BA, and aggregate) in a plastic bag for approximately 1 min;
(2) Mechanical mixing (Kitchenaid 5KSM95PSEGD) for 15 s at 58 rpm with the addition of distilled water;
(3) Manual mixing for approximately 45 s;
(4) Mechanical mixing for 75 s at 58 rpm.

All the prepared formulations were poured into metallic molds of 4 × 4 × 16 cm^3^ and vibrated for 2 min to remove possible air bubbles (according to the EN-196-1 standard [15]). Subsequently, the molds were placed inside a plastic bag for 24 h. Then, the samples were demolded and cured at 20 °C and 65% RH (climatic chamber). The most promising samples were further cured in distilled water at 20 °C (with water replacement every 7 days).

### 2.3. Raw Materials Characterization

The particle size distribution of the bottom ashes and commercial sand was obtained by sieving, using sieves with mesh sizes of 0.063, 0.125, 0.250, 0.500, 1, 2, and 3.15 mm (A7–A1). The particle size distribution of the BA fraction “<0.063 mm” and of the OPC was obtained by laser diffraction using a Coulter LS analyzer (LS 230 model, Brea, CA, USA). X-ray fluorescence (XRF, Phillips X’Pert PRO MPD spectrometer, Amsterdam, The Netherlands) was used to determine the chemical composition of the as-received BA, the 7 fractions obtained by the sieving process, and cement. The loss on ignition (LOI) at 1000 °C of the dried materials was also determined in a muffle. Thermal analyses (DTA/TG, differential, and thermogravimetric) of 2 fractions of the BA were performed simultaneously using STA 409 EP (NETZSCH, Selb, Germany). X-ray diffraction (XRD, Rigaku Geigerflex D/max-Series instrument, Tokyo, Japan) was used to evaluate the mineralogical composition of the samples. The phase identification was performed using the PANalytical X’Pert HighScore Plus PRO3 software version 4.7 (Almelo, The Netherlands). Additionally, scanning electron microscopy/energy dispersion spectroscopy (SEM/EDS, Hitachi SU-70, Tokyo, Japan, 25 kV acceleration voltage) was used to evaluate the microstructure of the BA.

### 2.4. Mortars’ Characterization

To assess the workability of the pastes in the fresh state, flow table tests were performed according to EN 1015-3 [16]. The water/binder (W/B) ratio used in each formulation was adjusted to maintain a constant final spread value of 140 ± 3 mm.

The hardened state properties of the mortars were evaluated after 7 and 28 days of curing. The compressive strength was measured using a Universal Testing Machine (AG-25TA-Shimadzu, Kyoto, Japan) with a displacement rate of 0.5 mm/min, in accordance with the standard EN 1015-11 [17]. The apparent density of the samples was determined by their weight and geometric volume, and the capillary coefficient was determined based on EN 1015-18 [18]. Durability tests (freeze–thaw resistance) were performed according to EN 998-2 [19]. For this, the specimens were dried in an oven at 50 °C for 24 h, immersed in water at room temperature for 24 h, and then frozen at −18 °C for 24 h, corresponding to one cycle, similar to [20]. This procedure was repeated 5, 15, and 25 times. Herein, the compressive strength, geometric density, and capillary index were evaluated. In all analyses, three replicates were tested, and the average and standard deviation error values were calculated.

## 3. Results and Discussion

### 3.1. Characterization of the Raw Materials

**Figure 2:**
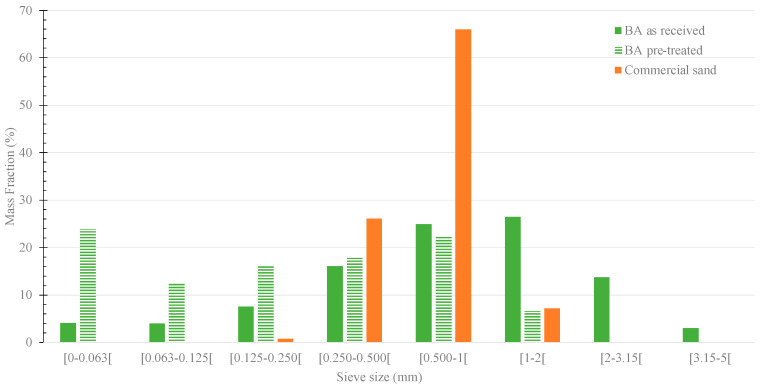
Granulometric distribution of the commercial sand and of the residue, as received and pre-treated (grinding and sieving at 2 mm).

The granulometric distributions of the commercial sand and BA, as received and pre-treated (ground and sieved), are presented in Figure 2.

The granulometric distribution of commercial sand ranges between 0.25 and 2 mm. As for the as-received residue, particles between 0.25 and 3.15 mm are predominant, accounting for approximately 84 wt.% of the material. In addition, the fraction below 63 μm was very low, comprising only 4 wt.%, which means that only a small part of the residue can be used as a substitute for OPC without further pre-treatment. However, after pre-treatment (grinding and sieving at 2 mm), the granulometric distribution changed significantly. Comparing the commercial sand with the pre-treated BA, some differences are still noticed, and the use of BA as aggregate might be compromised, but the content of the finer fraction significantly increased. The <63 μm fraction rose from 4 to 24 wt.%, and the fraction between 63 and 125 μm also showed an increase (from 4 to 12 wt.%). Thus, as expected, the milling increases the fraction of residue that can be used as a substitute for OPC or as a filler. However, this procedure has associated costs (e.g., energy consumption), especially during the milling process. Therefore, a simplified energy cost assessment was conducted, see Table 2, which also presents the estimation of the total energy required for the industrial pre-treatment processes of slag and BA. For the slag, a vertical roller mill was considered, and the slag specifications were humidity < 8% and Blaine (specific surface area) of 5 cm^2^/g [21]. This pre-treatment allows to reach a particle size lower than 60 μm, which enables the use of materials as a cement substitute or as filler. For the MSWI bottom ash, two scenarios were considered: Scenario 1—BA pre-treatment equal to that of the slag; Scenario 2—BA pre-treatment similar to that of the laboratory procedure: the industrial hammer mill and a vibrating screen for the sieving process. Note that the energy required was estimated considering the power and capacity of industrial machinery.

Analyzing Table 2, the estimated total energy required to reach a particle size lower than 60 μm is 45.2 kWh/ton for both the slag and BA (scenario 1). In scenario 2, the required energy is lower than that for the slag milling process, 37.7 vs. 45.2 kWh/ton. However, with this pre-treatment, only ~24 wt.% of BA is below 63 μm. Considering the energy cost, 0.09158 €/kWh [24], this increase corresponds only to an additional cost of ~0.70 €/ton. So, the pre-treatment selected should take into account the particle size needed for the BA valorization. Further, the additional cost of the pre-treatment process is below 5 €/ton, which is very appealing because the landfilling costs for this type of waste vary between 50 and 60 €/ton. Therefore, an agreement between the construction industry and the incineration unit could be made and would bring economic and environmental advantages with both scenarios if it is proven that BA could be used in mortar formulations.

BA exhibits a greyish color, and the presence of paper residues is only visible in the coarser fractions. However, contrary to what has been reported in other works [5,26], the presence of large inorganic material (ceramic, glass) was not observed, probably due to the sieving (5 mm) process conducted in the company.

**Figure 3:**
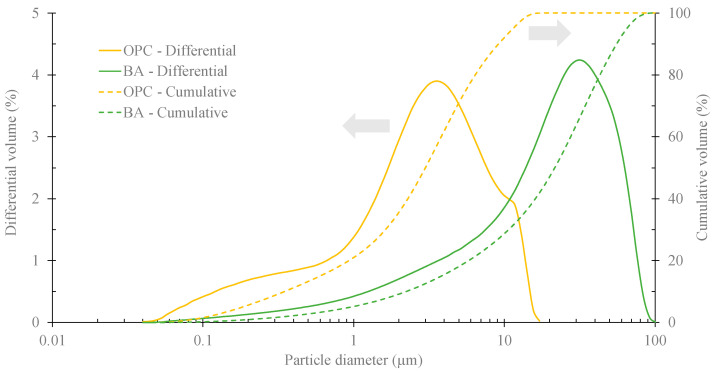
Granulometric distribution of OPC and pre-treated BA (<63 μm).

The granulometric distribution of OPC and the finer fraction (<63 μm) of pre-treated BA are shown in Figure 3. The average particle size of the cement is ≈4 µm, whereas that of BA is six times higher (25.5 μm). This significant difference is also reflected in the D50 and D90 values. The D50, which represents the mean diameter of the cement, is 3 μm, significantly lower than that of the ash (21.6 μm). Similarly, for the D90 value, the trend is consistent, with the cement having a value of 9.2 μm compared to the BA’s 55.8 μm.

**Figure 4:**
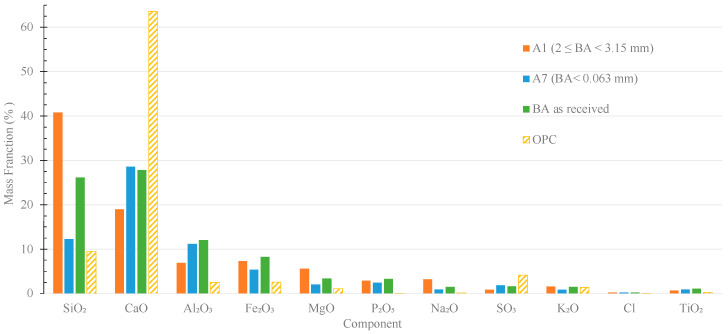
Chemical composition of OPC, BA as received, and fractions A1 and A7 obtained by sieving.

The chemical composition of A1 (2 ≤ ϕ < 3.15 mm) and A7 (<0.063 mm) fractions, as well as the as-received residue and the cement, were determined by XRF, and the main results are shown in Figure 4. The chemical composition of all fractions is presented in Table S1 (Supplementary Material).

The as-received residue and the fractions A1 and A7 contain SiO_2_, CaO, Al_2_O_3_, Fe_2_O_3_, MgO, P_2_O_5_, Na_2_O, SO_3_, K_2_O and TiO_2_. However, as shown in Figure 4, BA composition varies significantly with the particle size range. For instance, the SiO_2_ content considerably decreases with the reduction of the BA particle size (A1—42 wt.% and A7—12 wt.%), which is attributed to the greater amount of glass residues present in the coarser fraction. On the contrary, the trend is opposite, but less pronounced, for CaO, with the coarsest fraction (A1) containing 18 wt.% of calcium oxide and the finest (A7) having 28 wt.%. Similar trends were observed by Dou et al. [27] and Siddique [28] for MSWI ashes. For the other components present in the residue, the variation of their contents with the granulometry is less pronounced. As expected, OPC is much richer in CaO than the bottom ashes.

**Figure 5:**
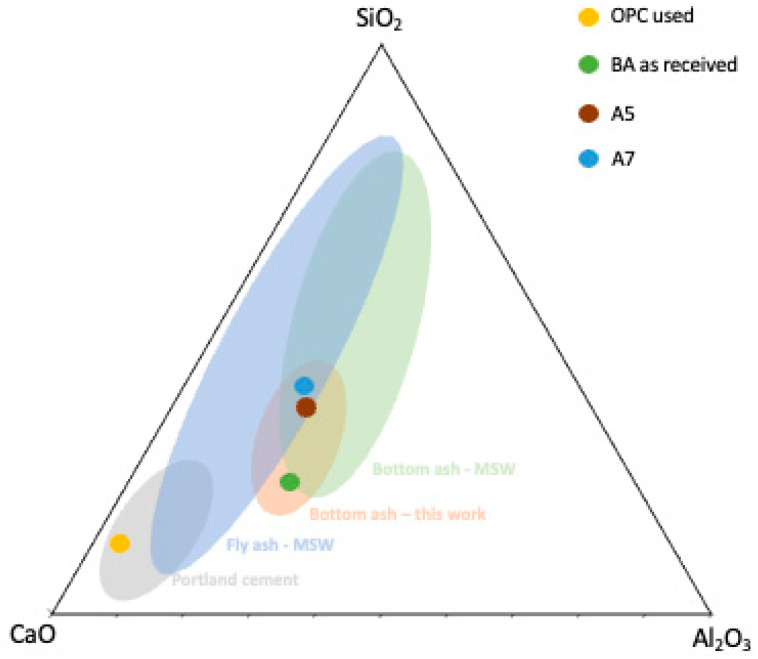
SiO_2_-CaO-Al_2_O_3_ ternary diagram with the compositions of the bottom and fly ash resulting from the burning of MSW, Portland cement II B/L 32.5N, BA as received, and two fractions (A5 and A7). Figure adapted from [29,30].

Figure 5 shows the ternary system SiO_2_–CaO–Al_2_O_3_ with the compositions of the raw materials used and the zones described in the literature for cement, MSW fly, and bottom ash [29,30]. From Figure 5, it can be observed that the different BA fractions have compositions that fall within the range of bottom ash from the burning of MSW. The composition of the as-received residue (green circle) is at the extreme end of the reported compositions. This may be due to the lack of chemical pre-treatments, which have been reported in other studies. Additionally, the location and time of waste collection, such as festive holidays, can also influence the BA composition.

To comply with the EN 197-1 standard [31], it is necessary to assess the contents of various constituents, namely chlorides, sulfur, and sodium. Table 3 lists the maximum values of the components referred to in the EN 197-1 standard and the values of the used BA factions. The sodium oxide equivalent (Na_equivalent_) was also assessed (Na_equivalent_ = Na_2_O + 0.658 K_2_O) [32].

For chlorine, the maximum permitted value is 0.1 wt.%, and the value present in the different fractions is 0.2 wt.%. Consequently, when BA is used as a partial substitute of cement or filler, for the maximum substitution level—30 *v*/*v* % (~13 wt.%), the chlorine content that it apport to the mixture will be only 0.03 wt.%. However, in the case of using BA as a partial or total substitute of the aggregate, the chlorine contents indicate that only 50 wt.% of BA should be used. Regarding the sulfate and sodium contents, the values are below the maximum established by the EN 197-1 standard, but higher values were obtained for the Na equivalent. In the case of partial substitution of cement or filler, no problems arise; however, in the case of aggregate substitution, the maximum quantity allowed should be approximately 60 wt.% (~70 *v*/*v* %), considering only the Na equivalent values. Nevertheless, to study the aggregate substitution by BA, 75 and 100 *v*/*v* % were also tested.

Loss on ignition (LOI) is an important parameter to consider when assessing the suitability of BA as a substitute for the binder. Table 4 shows the LOI values, at 1000 °C, for the residue fractions. It is observed that as the fraction size decreases, the LOI value increases. For instance, the LOI value for fraction A1 is 9.64 wt.%, while for A7 is 32.3 wt.% and 12.0 wt.% for the as-received BA. The EN 197-1 standard [31] specifies that the LOI of cement must be lower than 5 wt.%. Thus, based solely on the LOI value, the maximum replacement content of cement by BA from fraction A7 is approximately 15.5 wt.%. However, to study the binder substitution by BA, in this work, a maximum replacement of 30 *v*/*v* % was performed, which corresponds to ~13 wt.%.

Table 4 also includes the calculation of SiO_2_ + Al_2_O_3_ + Fe_2_O_3_ for different BA fractions. According to ASTM C618, a specification for coal fly ash and raw or calcined natural pozzolan for use in concrete), to be classified as type C, the fly ash needs to meet some chemical requirements, namely: SiO_2_ + Al_2_O_3_ + Fe_2_O_3_ > 50%; SO_3_ content < 5%; moisture content < 3% and LOI < 6% [33]. All BA fractions in this study meet the requirements for SO_3_ and moisture contents. However, none of the fractions meet the LOI requirement of <6% and only fraction A1 meets the SiO_2_ + Al_2_O_3_ + Fe_2_O_3_ requirement of higher than 50%. The as-received BA presents 46.41 wt.% for the sum of SiO_2_ + Al_2_O_3_ + Fe_2_O_3_ and an LOI value of 12.0%. Consequently, to be able to classify the BA as Class C and indicate that it may have hydraulic or binding behavior, the burning or trial/separation process of the Municipal Solid Waste (MSW) needs to be improved.

**Figure 6:**
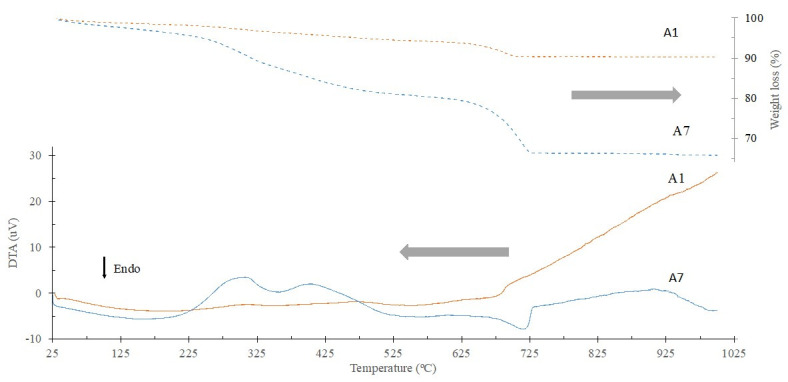
TG and DTA results of BA fractions A1 and A7.

The thermal gravimetric (TG) and differential thermal analyses (DTA) results for the BA fractions A7 and A1 are displayed in Figure 6, respectively. As shown in the thermal gravimetric results (dashed curves), the finer fraction (A7) exhibited a much higher weight loss (34.2 wt.%) compared to the coarser fraction (A1—9.8 wt.%), which is consistent with the LOI results. In the differential thermal analyses, an endothermic peak between 25 and 185 °C was observed in all fractions, accompanied by a weight loss due to dehydration. Between 185 and 640 °C, two exothermic peaks are observed. The first one, between 185 and 375 °C, was caused by the combustion of aliphatic compounds (long-chain compounds composed of carbon and hydrogen), and the second peak, between 375 and 650 °C, was due to the combustion of organic compounds, resulting in weight loss [34]. Finally, between 650 and 750 °C, there is an endothermic peak, which is related to the decomposition of carbonates and the release of CO_2_ (CaCO_3_ → CaO + CO_2_). This peak is also accompanied by a weight loss being more expressive in the finer fractions (12 wt.% for A7 versus 2 wt.% for A1), in accordance with their higher CaO contents determined by XRF [35].

**Figure 7:**
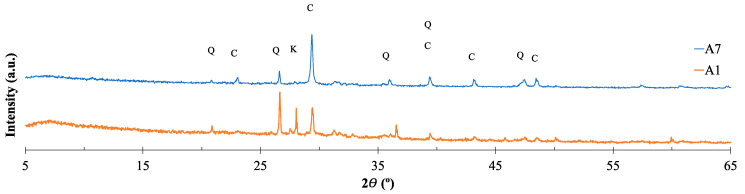
X-ray diffraction (XRD) patterns of the fractions of BA (Q—quartz, C—calcite, and K—microcline).

The crystalline phases present in the fractions A1 and A7 of BA were determined by X-ray diffraction (XRD), see Figure 7. The main crystalline phases detected were calcite (C) and quartz (Q). In the finer fraction, A7, the peak of quartz presents a lower intensity than in A1fraction, contrary to the calcite phase. These data are in agreement with the XRF results. Further, in the A1 fraction, the presence of microcline was also detected.

**Figure 8:**
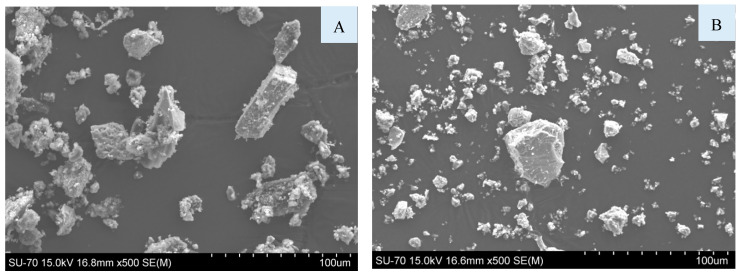
SEM micrographs of BA < 0.063 mm (**A**) and cement (**B**).

In Figure 8, it is possible to observe the SEM micrographs of the fraction A7 of the BA (A) and of the cement (B). The particle size of the BA is larger than that of the cement, corroborating the results of the granulometric distribution (Figure 3). BA and cement particles show heterogeneous sizes and shapes.

**Figure 9:**
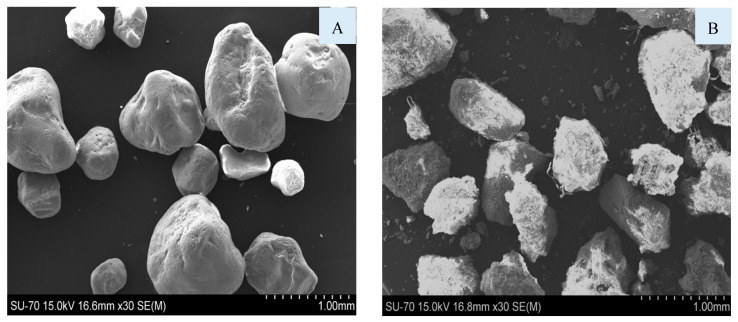
SEM micrographs of commercial sand (**A**) and BA [0.5, 2[ (**B**).

Figure 9 shows particles of commercial sand and a coarse fraction (0.5–2 mm) of BA. The two materials exhibit similar particle sizes; however, their surface is quite different. The sand particles are more spherical and rounded, while BA particles have rough surfaces and are less rounded, which can negatively affect the properties of the specimens once more water might be needed to lubricate the particles.

### 3.2. Characterization of the Mortars

#### 3.2.1. BA as Aggregate

**Figure 10:**
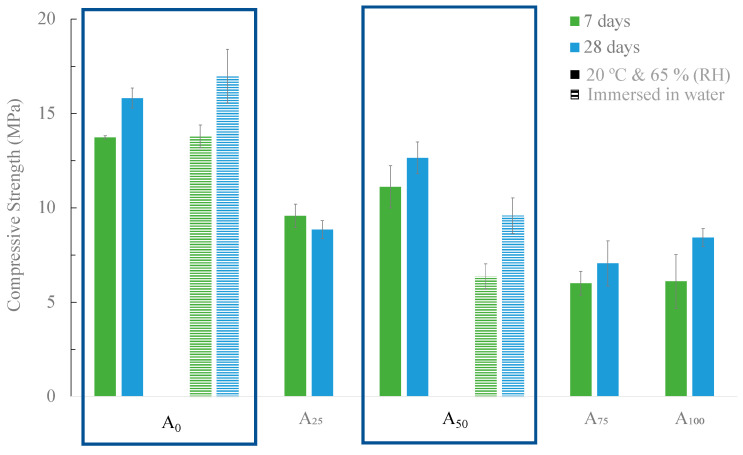
Compressive strength of the developed mortars in which BA was used as aggregate at 7 and 28 days of curing with two curing conditions: 20 °C and 65% RH and immersed in water.

The compressive strength values of the mortars with different substitution levels (from 0 to 100 wt.%) of commercial sand by BA are presented in Figure 10. The reference mortar and the most promising (A_50_) were cured under two different conditions: 20 °C and 65% RH and immersed in water. All the other samples were cured only in the climatic chamber (20 °C and 65% RH).

As shown in Figure 10, the increase in the substitution level deteriorated the mechanical resistance of the material. In the case of the curing in the climatic chamber (20 °C and 65% RH), the reference mortar (A_0_) exhibits a mechanical resistance of 15.82 MPa while mortars A_50_ and A_100_ exhibit 12.64 and 8.42 MPa corresponding to a reduction of ≈20 and 46%, respectively. This behavior may result from the increase in the amount of water used to prepare the samples with BA to maintain a constant spread value (140 mm). In the reference mortar, the water/binder ratio used was 0.6, while for the mortar with total replacement of commercial sand by BA (A_100_), the value was 0.83 (Table 1). The angular shape and surface roughness of BA might explain this tendency, as well as the presence of organic compounds. The increase in the water/binder ratio decreases the compressive strength of the materials [36]. In general, as expected, the samples exhibited an increase in compressive strength with a rise in curing time from 7 to 28 days [37].

**Figure 11:**
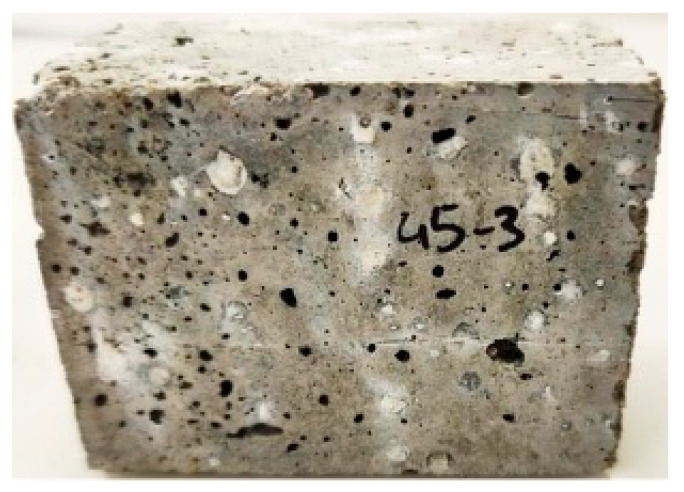
Efflorescence is present on the surface of the mortar A_50_.

Looking at the two different curing conditions, in the reference mortar (A_0_), a slight increase in the compressive strength was observed (from 15.82 to 16.98 MPa) when the materials were cured in water, bars with stripes. However, in the case of mortar A_50_, a decrease of approximately 25% in mechanical strength was observed, from 12.64 to 9.59 MPa. In fact, after 10 days of curing, efflorescence on the surface of the mortar A_50_ started to be noticed, as shown in Figure 11. The migration of the calcium present in the BA to the surface of the mortar explains that observation and might negatively affect the mechanical properties of the samples.

Table 5 presents the geometric density of the mortars cured in the climatic chamber at 7 and 28 days. When analyzing the effect of adding BA as aggregate, it was possible to verify that its incorporation caused a decrease in the density of the samples. This is due to the lower density of BA (1.30 g/cm^3^) compared to sand (1.81 g/cm^3^).

The difference in density between the A_0_ and A_100_ is notorious and also justifies the differences observed in the mechanical strength values. After 28 days of curing, the standard mortar (A_0_) has a density of 1.9 g/cm^3^ and the A_100_ 1.7 g/cm^3^. This decrease is in line with that reported in the literature, where studies whose focus was the replacement of aggregate by BA led to a decrease in density in samples with higher incorporation levels [38].

#### 3.2.2. BA as Partial Substitute of OPC

**Figure 12:**
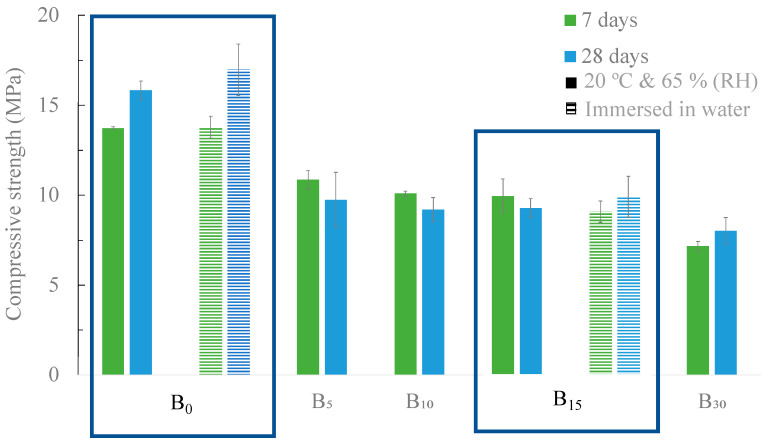
Compressive strength of the developed mortars in which OPC was substituted by BA at 7 and 28 days of curing with two curing conditions: 20 °C and 65% RH and immersed in water.

The compressive strength of the developed samples is shown in Figure 12, where two curing conditions were tested: climatic chamber (20 °C and 65% RH) and immersion in water.

The replacement of OPC with BA promotes a decrease in the mechanical strength of the samples for both curing conditions. The standard sample (B_0_), after 28 days of curing in the climatic chamber, exhibited a compressive strength of 15.8 MPa, and the replacement of 15 *v*/*v* % caused a decrease of approximately 40% (9.3 MPa). For lower substitution contents (5 and 10 *v*/*v* %), the values obtained are identical to the 15 *v*/*v* % replacement. An increase in the replacement content (30 *v*/*v* %) induced a greater decrease in the compressive strength (7.95 MPa). Nonetheless, all the developed materials are suitable for use in walls, as the minimum value accepted is 5 MPa [39]. These results are in line with those described by C. Lynn et al. [40] and N. Saikia et al. [41], who showed that replacing OPC with ash without any pre-treatment results in a decrease in compressive strength.

Table 6 presents the geometric density of the mortars cured in the climatic chamber. It can be seen that for both curing times, the geometric density of the samples decreased with the rise in the replacement level of OPC with BA. For example, after 28 days of curing, the standard mortar, B_0_, has a density of 1.9 g/cm^3^, while the sample B_30_ has a lower value, 1.8 g/cm^3^. This decrease can be attributed to the density difference between OPC and BA, 1.36 g/cm^3^ and 0.92 g/cm^3^, respectively. It can also result from the lower reactivity of the BA, producing less compact samples.

**Figure 13:**
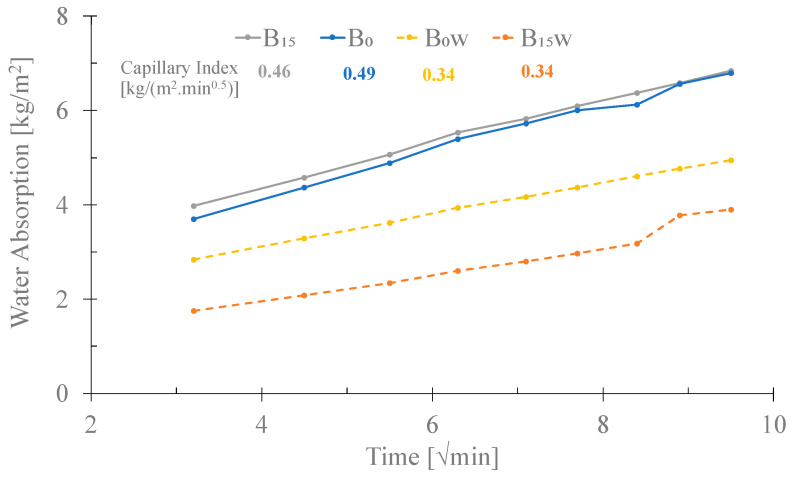
Influence of 15 *v*/*v* % BA in the water absorption of the mortars cured in the climatic chamber and immersed in water (w).

Figure 13 presents the data for the water absorption by capillarity of B0 and B15 samples cured in the climatic chamber and immersed in water (w). It is possible to verify that in the initial phase, the samples cured in water (B_x_w) have a lower water absorption value than the mortars cured in the climatic chamber. Similar behavior was observed for the capillary index values, which are smaller for the samples cured immersed in water (0.34 kg/m^2^.min.^0.5^). This might indicate that the mortars cured in water had lower porosity than those cured in the climatic chamber. Regarding the influence of the BA addition (15 *v*/*v* %), in the samples cured in water, no difference in the capillary index was observed. For the mortar cured in the climatic chamber, the obtained values are not equal but very closed (0.46 and 0.49 kg/m^2^.min.^0.5^). Nevertheless, all values are lower than those reported for mortars with OPC, which vary between 0.5 and 1.2 kg/m^2^.min.^0.5^ [42].

#### 3.2.3. BA as Filler

**Figure 14:**
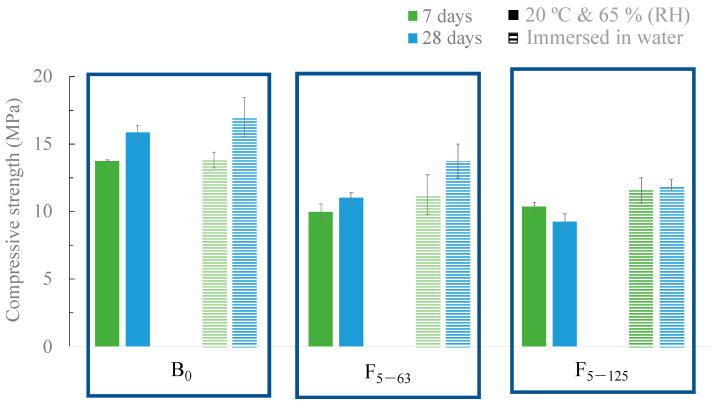
Compressive strength of the mortars with 5 wt.% of BA as filler after 7 and 28 days of curing at 20 °C and 65% RH and immersed in water.

The influence of the curing time (7 and 28 days) and conditions (climatic chamber and immersion in water) on the compressive strength are shown in Figure 14. In the case of samples cured in the climatic chamber (solid bars), the two particle sizes of BA tested as filler led to a decrease in the mechanical resistance of the samples. This fact is due to the presence of organic matter in the BA, which, over time, is going to create porosity and deteriorate the mortar properties, see TG in Figure 6. The standard mortar, B_0_, after 28 days of curing, exhibits a mechanical resistance of 15.8 MPa, while the mortars with BA as a filler (F_5–63_ and F_5–125_) exhibit 11.0 and 9.3 MPa, respectively. Similar behavior was observed for the samples cured and immersed in water (dashed bars). However, in all cases, the samples cured in water exhibited a slightly higher mechanical strength than those cured in the climatic chamber.

Table 7 presents the geometric densities of the mortars after 7 and 28 days of curing in the climatic chamber. The addition of BA as a filler causes a slight decrease (3 and 5%) in the density of the samples, which agrees with the compressive strength results. No significant variations were observed with the curing time. The decrease in the mortar’s properties when BA is used as a filler indicates that BA does not present a filler effect once it is not inert. Consequently, the municipal solid waste incineration ashes need to be pre-treated to be able to be used as a filler.

#### 3.2.4. Freeze–Thaw Tests

**Figure 15:**
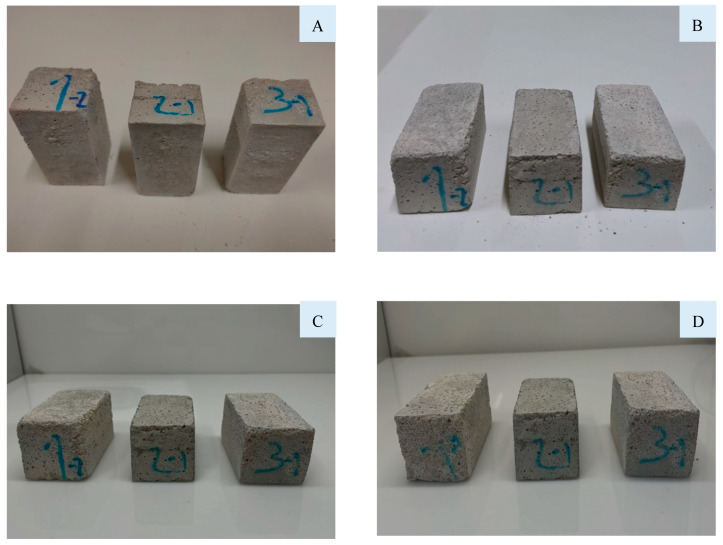
Mortars appearance after (**A**) 0 cycles (28 days of curing), (**B**) 5 cycles, (**C**) 15 cycles, and (**D**) 25 cycles (1—B_0_, 2—B_15_, and 3—F_5–63_).

From the previous results, the mortars B_0_, B_15_, and F_5–63_ were selected to evaluate the durability of freeze–thaw cycles. Figure 15 shows the appearance of the samples after 28 days of curing, 5, 15, and 25 cycles. Macroscopically, the mortars did not show any difference, neither degradation of the surface nor the loose aggregates or cracks.

**Figure 16:**
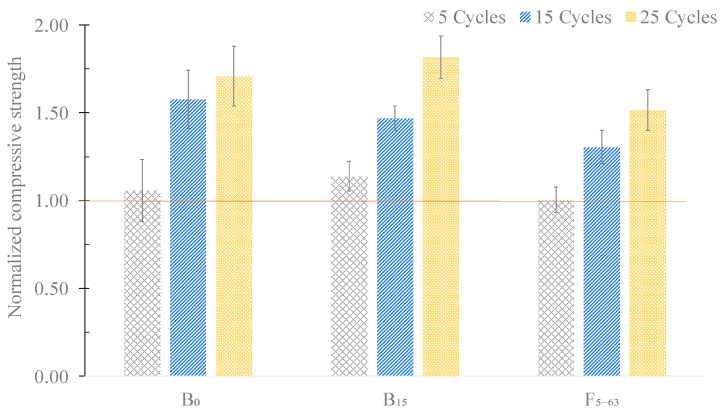
Effect of the freeze–thaw cycles on the compressive strength of the mortars B_0_, B_15_, and F_5–63_.

The effect of the freeze–thaw cycles on the compressive strength of the mortars are shown in Figure 16. Herein, a normalization factor was considered, corresponding to the strength of the mortars at 28 days of curing.

As shown in Figure 16, in all the tested specimens, the compressive strength increased after being subjected to 25 cycles, indicating that all mixtures can safely resist freeze and thaw deterioration. In fact, the reference mortars exhibited an increase of approximately 71% in mechanical strength, sample B_15_ 82%, and F_5–63_ 52%, when compared to the mechanical strength at 28 days (without any freeze–thaw cycle).

Table 8 presents the geometric density of the mortars after 28 days of curing, 5, 15, and 25 freeze–thaw cycles. In all samples, no change was observed in the geometric density with the cycles.

**Figure 17:**
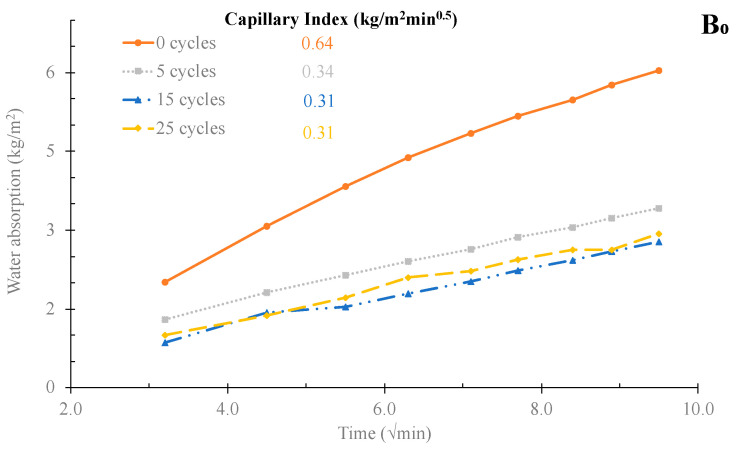

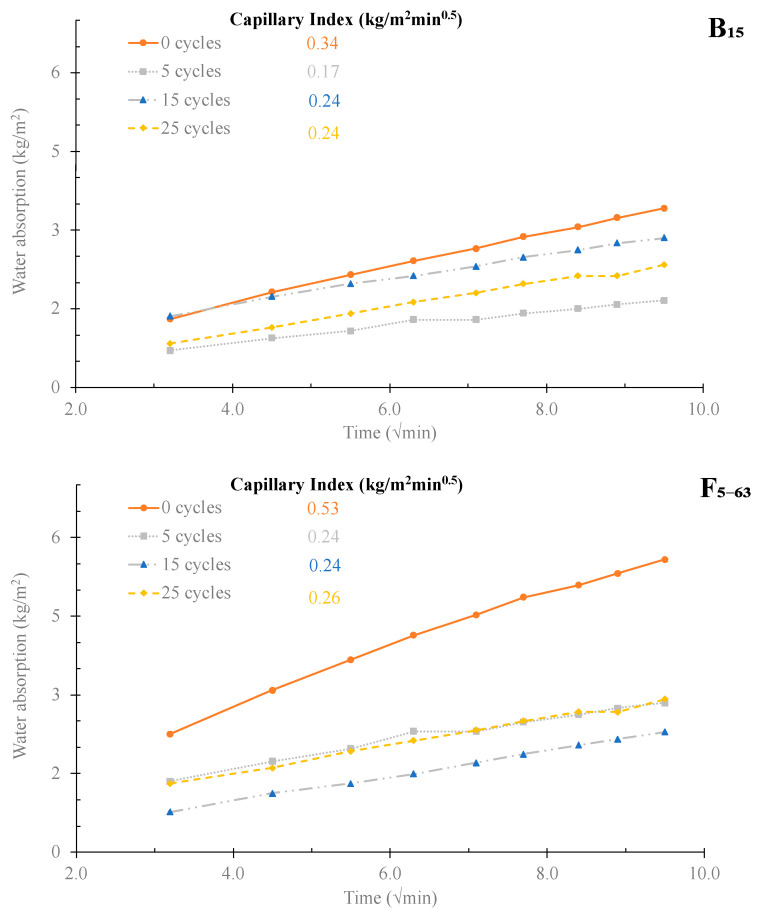
Effect of the freeze–thaw cycles on the water absorption by capillary of the mortars B_0_, B_15_, and F_5–63_.

The water absorption by capillary results are shown in Figure 17. It can be observed that, in all tested samples, the water absorption and the capillary index of the mortars after 28 days of curing (0 cycles) were always higher than the values exhibited by the mortars subjected to the freeze–thaw tests. This might indicate that the mortars, after 28 days of curing, had higher porosity, which is also aligned with the compressive strength results. Small differences were observed between the number of cycles; however, the samples B_0_, B_15_, and F_5–63_ present a lower capillary index than the reference, 0.24, 0.26, and 0.31 kg/m^2^.min.^0.5^, respectively, for mortars with 25 freeze–thaw cycles. Consequently, the mortars with 15 *v*/*v* % of BA or with 5 *v*/*v* % of BA as filler show excellent frost resistance.

## 4. Conclusions

Mortars with Municipal Solid Waste bottom ashes were developed in this study. Three approaches of using BA were tested together with several incorporation/substitution levels: (i) aggregate (0 to 100 *v*/*v* %), (ii) partial binder substitute (0 to 30 *v*/*v* %), and (iii) filler (5 *v*/*v* % with two particle sizes <63 and <125 μm).

BA has smaller particles than commercial sand, but its surface is more heterogeneous and rougher. The fraction below 63 μm presents an average particle size much higher (≈six times) than that of the cement, 25.5 and 4 μm, respectively. Analyzing the chemical composition and loss on ignition of bottom ash (BA) yielded crucial data on potential replacement levels for different applications, suggesting a viable aggregate replacement at ~70 *v*/*v* % and a binder substitution at around 30 *v*/*v* %.

Hardened state properties of the mortars allowed us to conclude that:

- BA as an aggregate leads to a decrease in the compressive strength of the mortars, and efflorescence appears on the sample surface.
- BA as a binder (30 *v*/*v* %) also led to a decrease in the compressive strength of the mortars, from 15.8 to 8.0 MPa. However, the capillary indexes of the developed mortars are lower than those reported for OPC mortars, and these materials are suitable for use in walls.
- BA as filler presented a slight decrease in the mechanical resistance. It was more pronounced for the higher particle size fraction—11.0 and 9.3 MPa with 63 and 125 μm, respectively.
- No significant influence of the two tested curing conditions, immersed in water and in a climatic chamber (20 °C and 65% RH), was observed.
- Mortars with 15 *v*/*v* % of BA as binder and 5 *v*/*v* % of BA as filler presented excellent resistance in the freeze–thaw durability tests for up to 25 cycles.

However, municipal solid waste incineration bottom ash presents some limitations to be used as received, either as OPC replacement or as filler, namely due to its chemical composition, such as the presence of organic matter the chlorine content that deteriorates and negatively affects the mortar’s properties. Therefore, pre-treatments are needed to allow the use of this waste. Nonetheless, the developed mortars, namely, 15 *v*/*v* % of BA as binder and 5 *v*/*v* % of BA as filler, are suitable for use as building material and wall construction. Moreover, the use of MSWI ash in the preparation of mortars can reduce its landfilled quantity and contribute to the decarbonization of the construction industry, being aligned with the Net Zero Emissions scenario.

## Figures and Tables

**Table 1:** Compositions of the prepared dense mortars.

		Mass (g)				Ratio			
Sample	BA (*v*/*v* %)	Commercial Sand	BA as Aggregate	BA as Binder/Filler	OPC	Water/ Binder W/B	Aggregate/ Binder A/B (vol.)	A/B (wt.)	Final Spread Values (mm)
A₀	0	905.0	0	-	226.7	0.60	3.0	4.0	140
A₂₅	25	679.0	162.5			0.62		3.7	140
A₅₀	50	452.5	325.0			0.67		3.4	139
A₇₅	75	226.3	487.5			0.82		3.2	141
A₁₀₀	100	-	650.0			0.83		2.9	141
B₀	-	905.0	-	-	226.7	0.60	3.0	4.0	140
B₅	5			4.6	219.9	0.57		4.0	141
B₁₀	10			9.2	213.1	0.61		4.0	143
B₁₅	15			13.8	206.3	0.56		4.1	141
B_30_	30			27.6	185.9	0.59		4.2	140
F_5–63_	5	905.0	-	4.6	226.7	0.60	2.9	3.9	140
F_5–125_									

**Table 2:** Estimation of the total energy required and cost for the industrial pre-treatment processes for slag and BA.

	Process Step\Material	Slag	BA	
			Scenario 1	Scenario 2
Energy required (kWh/ton)	Vertical Roller Mill [21]	45.2 ^a^	45.2 ^a^	-
	Sieve [22]	-	-	1.1 ^b^
	Hammer mill [23]	-	-	36.6 ^c^
Estimated total energy required (kWh/ton)		45.2	45.2	37.7
Estimated energy cost (€/ton) ^d^		4.14		3.45

^a^ Total electric power of vertical roller mill for slag with a maximum humidity of 8% and with a specific surface area (Blaine) of 5 cm^2^/g [21]; ^b^ Power = 11.00 kW; capacity 10 t/hour [22]; ^c^ Power = 110–160 kW capacity 3–6 ton/h [23]; ^d^ Energy cost of 0.09158 €/kWh [24,25].

**Table 3:** Maximum content of various constituents (SO_3_ and Na) according to the EN 197-1 standard and values of the used BA fractions.

	Maximum Value (wt.%)	A7 (<0.063 mm)	A6 (0.063 ≤ ϕ < 0.125 mm)	A2-A3 (0.5 ≤ ϕ < 2 mm)
SO_3_	3.5 [33]	1.9	1.8	1.4
Na_2_O	1.5	0.9	1.0	1.7
Na_equivalente_	1.5	1.5	1.6	2.6

**Table 4:** Loss on ignition and sum of SiO_2_ + Al_2_O_3_ + Fe_2_O_3_ of the BA fractions.

BA Fraction	A7 [0, 0.063[	A6 [0.063, 0.125[	A5 [0.125, 0.25[	A4 [0.25, 0.5[	A3 [0.5, 1[	A2 [1, 2[	A1 [2, 3.15[	BA as Received
**Loss on ignition** **(wt.%)**	32.34	26.33	20.66	19.56	19.51	14.59	9.64	11.95
SiO_2_ + Al_2_O_3_ + Fe_2_O_3_ * **(wt.%)**	28.66	33.02	36.62	41.61	42.26	48.08	54.94	46.41

* min 50% according to ASTM C618 for class C coal ash.

**Table 5:** Geometric density of mortars with different contents of BA as aggregate with 7 and 28 days of curing in a climatic chamber.

Samples	Density (g/cm^3^)	
	7 Days	28 Days
A_0_	1.9 ± 0.1	1.9 ± 0.1
A_25_	1.9 ± 0.1	1.8 ± 0.1
A_50_	1.8 ± 0.1	1.8 ± 0.1
A_75_	1.7 ± 0.1	1.7 ± 0.1
A_100_	1.6 ± 0.1	1.7 ± 0.1

**Table 6:** Geometric density of mortars in which OPC was partially substituted by BA after 7 and 28 days of curing in the climatic chamber.

Samples	Density (g/cm^3^)	
	7 Days	28 Days
B_0_	1.9 ± 0.1	1.9 ± 0.1
B_5_	1.9 ± 0.1	1.9 ± 0.1
B_10_	1.9 ± 0.1	1.9 ± 0.1
B_15_	1.8 ± 0.1	1.9 ± 0.1
B_30_	1.8 ± 0.1	1.8 ± 0.1

**Table 7:** Geometric density of mortars with 5 wt.% of BA as filler after 7 and 28 days of curing in a climatic chamber.

Samples	Density (g/cm^3^)	
	7 Days	28 Days
B_0_	1.9 ± 0.1	1.9 ± 0.1
F_5–63_	1.8 ± 0.1	1.9 ± 0.1
F_5–125_	1.8 ± 0.1	1.8 ± 0.1

**Table 8:** Effect of the freeze–thaw cycles on the geometric density of the mortars B_0_, B_15_, and F_5–63_.

Samples	Density (g/cm^3^)			
	28 Days	5 Cycles	15 Cycles	25 Cycles
B_0_	1.9 ± 0.1	2.0 ± 0.1	1.9 ± 0.1	2.0 ± 0.1
B_15_	1.9 ± 0.1	1.9 ± 0.1	1.9 ± 0.1	2.0 ± 0.1
F_5–63_	1.9 ± 0.1	2.0 ± 0.1	2.0 ± 0.1	1.9 ± 0.1

## Data Availability

Not applicable.

## Supplementary Materials

The following supporting information can be downloaded at: https://www.mdpi.com/article/10.3390/ma16216933/s1, Table S1: Chemical composition of BA as received and of the seven fractions obtained by sieving (A1–A7).
