# Supplementary material for: Development of Eco-Mortars with the Incorporation of Municipal Solid Wastes Incineration Ash
Source: Materials (Basel). 2023 Oct 28;16(21):6933. doi: 10.3390/ma16216933 (PMC10649002; doi:10.3390/ma16216933)
Supplement: Supplementary file 1 [file materials-16-06933-s001.zip › materials-2643671-supplementary.pdf]

Supplementary Material

Table S1. - Chemical composition of BA as received and of the seven fractions obtained by sieving (A1-A7).

Component	A1	A2	A3	A4	A5	A6	A7	BA as received
SiO_2	40.79	30.33	22.75	21.22	16.53	14.55	12.24	26.14
CaO	18.96	22.19	25.01	26.38	30.36	29.22	28.59	27.76
Al_2O_3	6.88	9.63	12.01	12.44	12.27	12.12	11.12	12.00
Fe_2O_3	7.27	8.12	7.50	7.94	7.83	6.35	5.29	8.27
MgO	5.54	4.63	3.15	2.44	2.30	2.16	1.98	3.33
P_2O_5	2.90	3.38	3.13	3.40	3.20	2.96	2.41	3.24
Na_2O	3.21	1.90	1.41	1.24	1.17	1.02	0.91	1.49
SO_3	0.80	1.25	1.64	1.68	1.94	1.84	1.88	1.63
K_2O	1.51	1.56	1.33	1.25	1.05	0.91	0.86	1.43
Cl	0.16	0.19	0.19	0.20	0.19	0.18	0.17	0.19
TiO_2	0.70	0.84	0.97	0.95	1.10	0.97	0.92	1.08
